# Case Report: Concurrent Fabry disease, IgA nephropathy and undifferentiated connective tissue disease in a female with normal enzyme activity

**DOI:** 10.3389/fimmu.2026.1945437

**Published:** 2026-09-17

**Authors:** Aoteng Cui, Sichao Ma, Tingting Sun, Mingxin Chang, Sirui Lu, Shaojie Fu, Hongzhao Xu

**Affiliations:** 1 Department of Nephrology, The First Hospital of Jilin University, Changchun, China; 2 Department of Nephrology, The Affiliated Hospital of Changchun University of Chinese Medicine, Changchun, China; 3 The First Norman Bethune Clinical College, Jilin University, Changchun, China

**Keywords:** case report, Fabry disease, globotriaosylceramide, IgA nephropathy, immune dysregulation, lysosomal storage disease, undifferentiated connective tissue disease

## Abstract

**Rationale:**

The diagnosis of Fabry disease (FD) in heterozygous females remains clinically challenging, as random X-chromosome inactivation frequently leads to preserved α-galactosidase A (α-Gal A) activity and absence of classic clinical stigmata. While concurrent FD and IgA nephropathy (IgAN) is a rare comorbidity predominantly reported in East Asian females, its overlap with systemic autoimmune conditions such as undifferentiated connective tissue disease (UCTD) has not been documented. The shared immunopathogenic mechanisms linking lysosomal sphingolipid storage, immune-mediated glomerular injury, and systemic autoimmunity remain poorly understood.

**Patient concerns and diagnoses:**

A 44-year-old Chinese woman presented with facial and lower limb edema, proteinuria (2.772 g/24h), and microscopic hematuria. She lacked classic FD manifestations, and both plasma α-Gal A activity (4.31 μmol/L/h) and lyso-Gb3 (0.52 ng/mL) were normal. Renal biopsy confirmed IgAN (Lee grade III, M1E1S1T0-C0) but electron microscopy incidentally identified pathognomonic lamellated zebra bodies in podocytes. GLA gene sequencing identified a heterozygous c.593T>C (p.Ile198Thr) mutation, confirming FD. Serologic workup revealed high-titer ANA (1:1000) and anti-SSA positivity, fulfilling UCTD criteria.

**Outcomes:**

The patient declined enzyme replacement therapy and received renoprotective regimens combined with hydroxychloroquine. At 6-month follow-up, renal function remained stable and no new UCTD manifestations occurred. Family screening identified her son as a hemizygous GLA carrier.

**Conclusion:**

This case highlights that routine electron microscopy during renal biopsy is critical for detecting occult FD in females with normal enzyme activity, and is consistent with a biologically plausible hypothesis of Gb3-driven immune dysregulation as a potential shared link among FD, IgAN, and systemic autoimmunity, which requires further validation in larger cohorts and functional studies.

## 1 Introduction

Fabry disease (FD) is an X-linked lysosomal storage disorder caused by pathogenic mutations in the GLA gene, which leads to deficient α-galactosidase A (α-Gal A) activity and progressive globotriaosylceramide (Gb3) deposition. Due to random X-chromosome inactivation, heterozygous females often present with preserved enzyme activity and absent classic stigmata, resulting in frequent underdiagnosis (1, 2). Emerging evidence indicates that Gb3 accumulation drives innate immune activation, and autoantibody positivity is common in FD patients, suggesting an intrinsic link between lysosomal storage and immune dysregulation (3, 4).

IgA nephropathy (IgAN) is the most common primary immune-mediated glomerulonephritis worldwide. Concurrent FD and IgAN is extremely rare, with most reported cases from East Asian female populations, and the shared immunopathogenesis remains largely unclear (5, 6). The overlap of FD with systemic autoimmune conditions such as undifferentiated connective tissue disease (UCTD) has scarcely been documented, and no triple comorbidity of FD, IgAN and UCTD has been reported to date.

Here we report a rare case of concurrent FD, IgAN and UCTD in a 44-year-old Chinese female with normal plasma α-Gal A activity, whose FD diagnosis was incidentally detected by renal biopsy electron microscopy. This case highlights the diagnostic value of routine electron microscopy for occult FD, and raises the possibility of shared Gb3-driven immune dysregulation across lysosomal storage disease, glomerulonephritis, and systemic autoimmunity, which remains to be further investigated.

## 2 Case presentation

A 44-year-old female was admitted on November 4, 2024, with a 3-year history of renal impairment and one week of progressive facial and lower extremity edema. Three years earlier, impaired renal function was incidentally detected during a routine health check, and glomerulonephritis was suspected clinically; however, she declined renal biopsy for definitive diagnosis. She denied nephrotoxin exposure, alcohol use, and smoking. She had no history of hypertension prior to admission. She received self-administered traditional Chinese medicine (TCM) therapy containing herbal ingredients including Abelmoschus manihot, Pueraria lobata, and Poria cocos, but achieved no satisfactory therapeutic response, with persistent progression of renal impairment. The exact dosage, formulation, and duration of each herbal ingredient were not documented in the medical records, as the preparations were obtained without a formal prescription from a TCM physician; the patient was unable to recall the precise doses upon follow-up. No adverse effects attributable to these herbal preparations were reported.

On admission, blood pressure was 160/99 mmHg. Physical examination revealed height 162 cm, weight 58 kg (BMI 22.1 kg/m²), heart rate 78 bpm, and temperature 36.5 °C. No angiokeratoma, corneal verticillata, rash, or joint swelling was observed. Mild pitting edema of the face and bilateral lower extremities was present, with no organomegaly or neurological abnormalities. Family history was negative for renal disease, Fabry disease, and connective tissue disease in her parents and siblings; her father was deceased of unknown cause and her mother was alive without major medical conditions. Notably, her son was subsequently identified as a carrier of the GLA mutation.

Laboratory tests on admission showed elevated serum creatinine of 79.2 μmol/L (reference range 41–73 μmol/L), decreased serum albumin of 34.3 g/L (reference range 40.0–55.0 g/L), and 24-hour urinary protein excretion of 2.772 g (reference range <0.15 g). Urinalysis revealed proteinuria (3+), hematuria (3+), and 490.70 erythrocytes/μL (reference range 0–23/μL). Serum IgG was slightly reduced at 8.06 g/L (reference range 8.6–17.4 g/L). Antinuclear antibody (ANA) testing showed both centromere pattern (+++) and granular pattern (+++) at a titer of 1:1000, with equivocal anti-Scl-70 (±) and strongly positive anti-SSA-52/Ro52 (++). Other routine laboratory parameters were unremarkable. Urinary system ultrasound demonstrated normal morphology and size of both kidneys (right kidney: 103 mm × 36 mm; left kidney: 98 mm × 43 mm), with bilateral renal cysts (multiple cysts in the right kidney, largest 22 mm × 20 mm; one cyst in the left kidney, 5 mm × 5 mm). Echocardiography only indicated mild mitral regurgitation, with no ventricular wall thickening (**Supplementary Table 1**).

On November 6, 2024, the patient underwent ultrasound-guided renal biopsy. Light microscopy identified 13 sampled glomeruli, 2 of which showed global sclerosis. The remaining glomeruli had moderately open capillary loops, with diffuse mild mesangial cell and matrix hyperplasia that was more prominent in segmental and focal regions. Segmental endothelial cell swelling with mild hyperplasia was observed, 3 glomeruli showed capsular adhesion, and 2 had segmental sclerosis. The glomerular basement membrane showed mild segmental thickening, with a small amount of eosinophilic deposits in the mesangial area and focal inflammatory cell infiltration. Renal tubular epithelial cells exhibited vacuolar degeneration with visible protein casts and focal mild tubular atrophy. Focal renal interstitial infiltration of lymphocytes and macrophages was accompanied by mild fibrosis, and no obvious abnormalities were found in small arteries. Congo red staining was negative. Immunofluorescence analysis of 6 glomeruli showed granular mesangial deposition of IgA (++++), IgM (+++), C3 (++), kappa light chain (++++), and lambda light chain (++++), with negative staining for IgG, C4, C1q, fibrinogen, and IgG subclasses (IgG1–IgG4). These findings were consistent with focal proliferative IgA nephropathy (Lee classification: Grade III; Oxford classification: M1E1S1T0-C0). Electron microscopy revealed mesangial electron-dense deposits and diffuse podocyte foot process effacement, consistent with IgAN with secondary focal segmental glomerulosclerosis (FSGS)-like lesions (**Figure 1**). Notably, lamellated myeloid (zebra) bodies were identified within podocytes, raising suspicion for Fabry disease.

**Figure 1:**
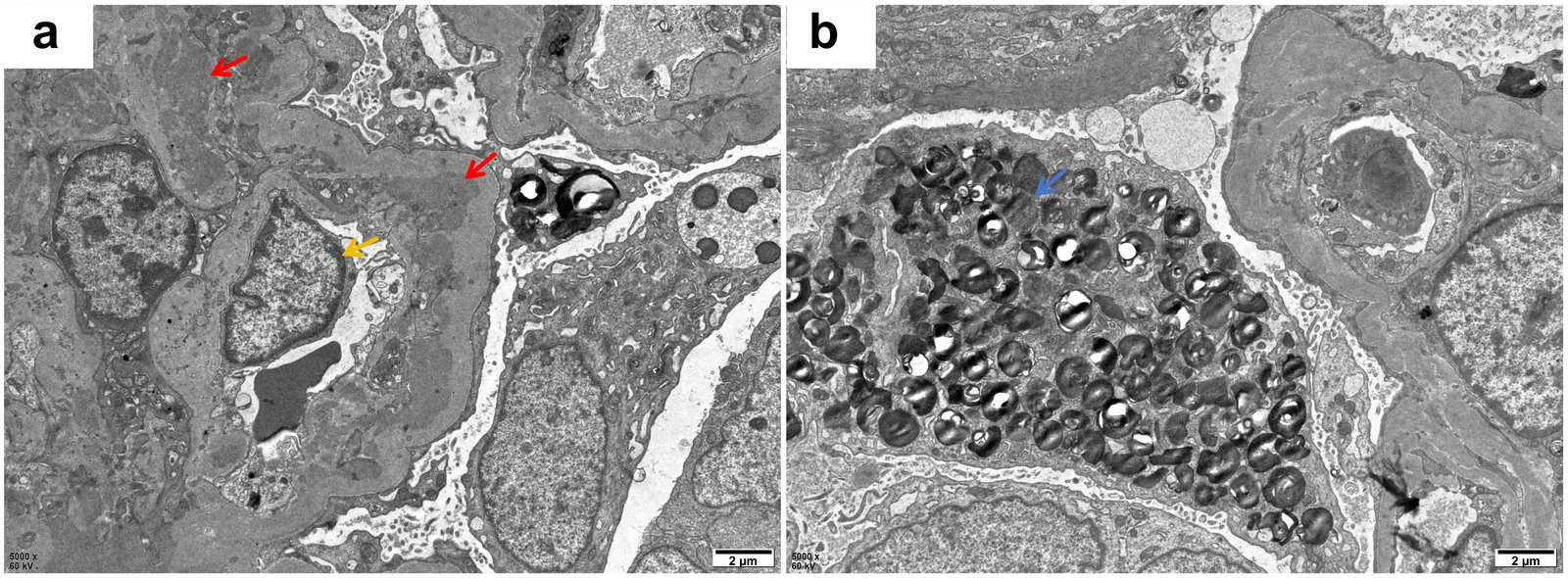
Renal pathological findings. **(a)** Electron microscopy shows mesangial electron-dense deposits (red arrows) and diffuse podocyte foot process effacement (yellow arrow), consistent with IgA nephropathy. **(b)** Abundant lamellated zebra bodies (blue arrow) and multilayered myelin-like glycosphingolipid inclusions (black arrow) within podocytes, pathognomonic for Fabry nephropathy. Scale bars: 2 μm.

To verify the suspected diagnosis of FD, systematic biochemical and genetic testing was performed. Plasma α-Gal A activity was 4.31 μmol/L/h (reference range: 2.20–17.65 μmol/L/h), and plasma lyso-Gb3 level was 0.52 ng/mL (reference range: <1.11 ng/mL), both within normal limits. Given the high pathological suspicion of FD, GLA gene sequencing was conducted, which identified a heterozygous pathogenic missense mutation NM_000169.3:c.593T>C (p.Ile198Thr), confirming the diagnosis of FD (**Figure 2**). The patient had no classic systemic manifestations of FD, including neuropathic pain, exercise intolerance, gastrointestinal symptoms, hypohidrosis, or angiokeratoma, consistent with an atypical mild phenotype in a heterozygous female. Additional comorbidities confirmed during hospitalization included stage 2 hypertension, hyperlipidemia, and bilateral renal cysts.

**Figure 2:**
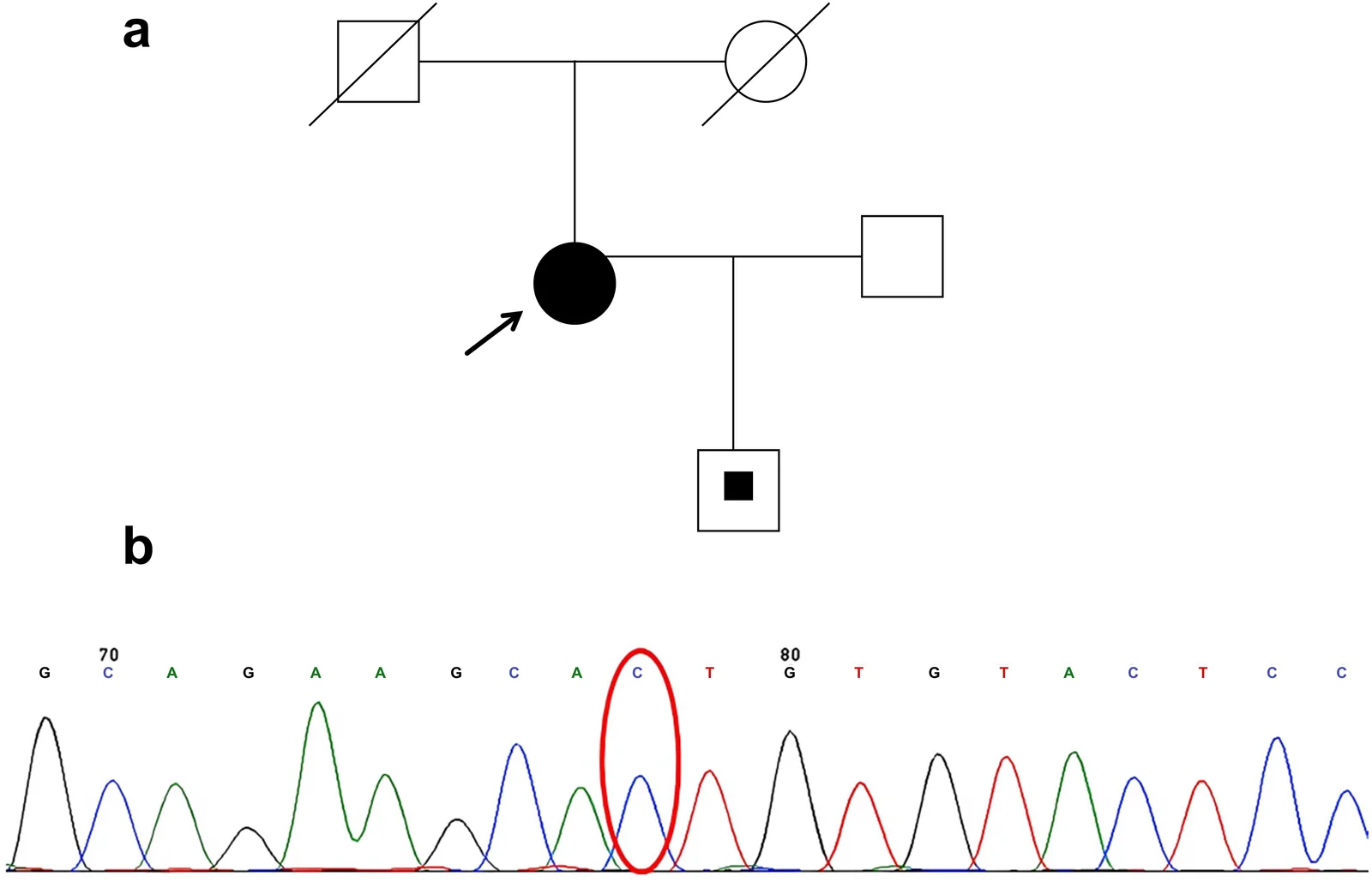
Pedigree chart and genetic verification. **(a)** The box represents male, the circle represents female, the arrow represents proband, the slash indicates deceased, the black filling indicates clinical involvement (symptomatic or organ involvement), and the dot at the center of the box represents asymptomatic gene carriers. **(b)** The genetic verification map of the patient’s son chrX:100655700 C.593T >C hemizygous variation.

After discharge, the patient was referred to the Department of Rheumatology and Immunology for systematic autoimmune evaluation. Salivary gland function assessment via 99mTc-pertechnetate static and dynamic imaging showed normal uptake and excretion function of bilateral salivary glands. Ophthalmic examination revealed tear secretion of 19 mm/5 min in the right eye and 18 mm/5 min in the left eye (normal >15 mm/5 min), and tear film break-up time (TBUT) of 3 s bilaterally (normal >10 s), with positive corneal epithelial staining. These findings met the diagnostic criteria for moderate dry eye syndrome (2017 DEWS II criteria). The patient had positive high-titer ANA and exocrine gland involvement, but did not meet the 2019 ACR/EULAR classification criteria for primary Sjögren’s syndrome, nor the classification criteria for other well-defined connective tissue diseases. Therefore, a diagnosis of UCTD was established according to the criteria proposed by Mosca et al. (7), based on moderate dry eye syndrome as a clinical manifestation of connective tissue disease, high-titer ANA (1:1000) with strongly positive anti-SSA-52/Ro52, a disease duration of at least 3 years, and the failure to meet classification criteria for any defined connective tissue disease. The diagnosis was confirmed by rheumatology consultation (**Figure 3**).

**Figure 3:**
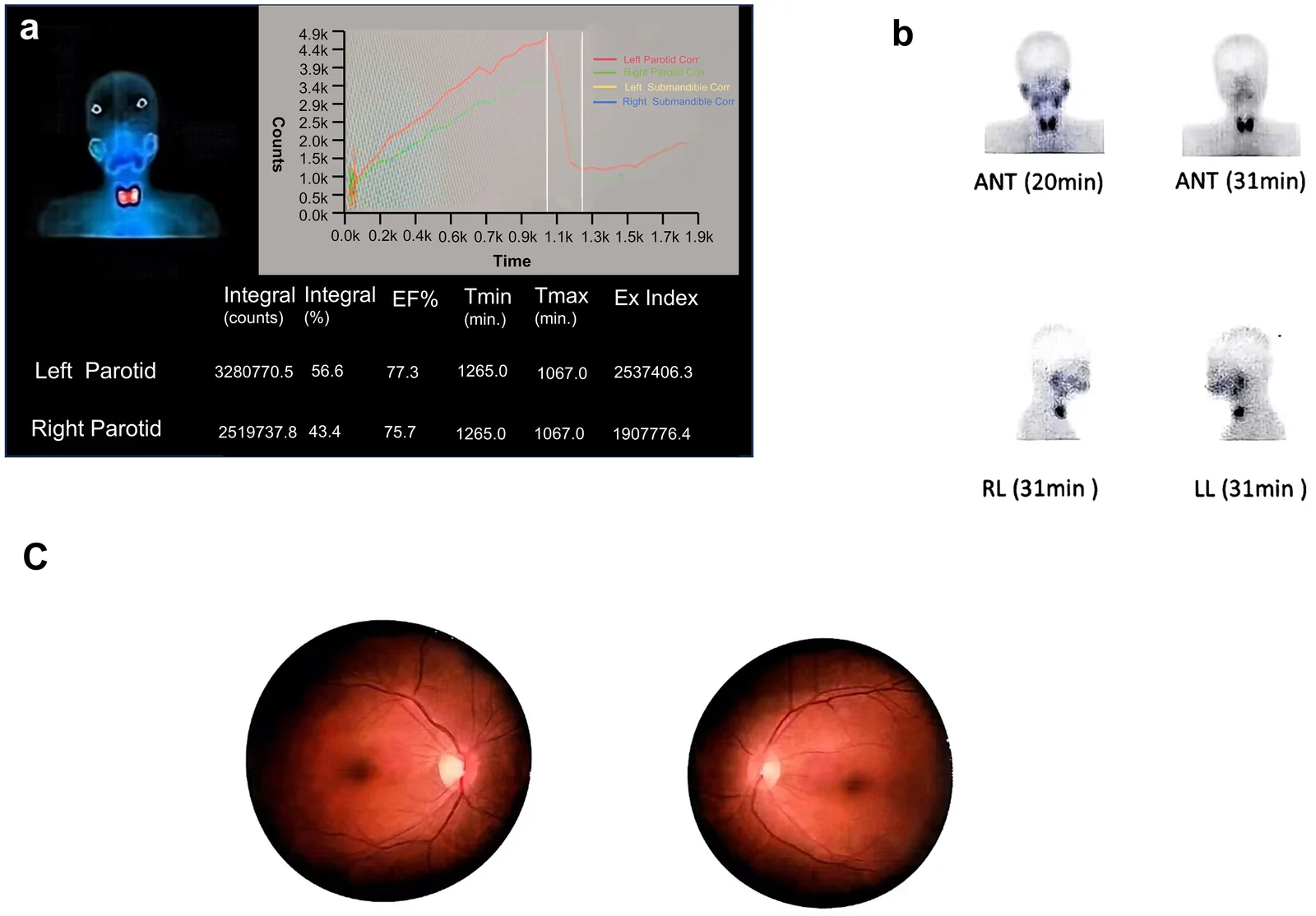
Examinations related to rheumatic and autoimmune diseases. **(a)** Dynamic imaging of the patient’s salivary glands. Blood flow imaging: the blood perfusion of bilateral salivary glands is basically normal. Blood pool imaging: Imaging of bilateral salivary glands within 20 minutes after injection. Twenty-one minutes after the injection, oral vitamin C was administered, and significant secretion was observed in both salivary glands. Curve graph: the blood flow segments, uptake segments and excretion segments of the bilateral salivary glands are basically normal. **(b)** Static imaging of salivary glands. Twenty minutes after intravenous injection of 99mTcO4-185MBq, the bilateral salivary glands gradually became clear, and their positions, sizes, shapes and radioactive distributions were basically normal. Oral administration of vitamin C was given 21 minutes after injection. At 3 minutes, it was observed that the radioactive distribution of bilateral salivary glands was significantly reduced compared with before. **(c)** Ophthalmoscopy examination. The boundaries of both optic discs are clear, with a light red color. The retina is orange-red, and the proportion of vascular course is roughly normal. The fovea centralis of the macula reflects light in clearly.

A comprehensive treatment regimen was formulated: sacubitril/valsartan 50 mg once daily for blood pressure control and renoprotection; dapagliflozin 10 mg once daily combined with finerenone 20 mg once daily for proteinuria reduction; atorvastatin 10 mg once daily for lipid-lowering therapy; and adjuvant renal supportive therapy with branched-chain amino acid supplementation. After 3 months of treatment, the patient’s serum creatinine increased transiently to 101.1 μmol/L. She voluntarily discontinued conventional Western pharmacotherapy and switched back to traditional Chinese medicine, and her serum creatinine subsequently stabilized at around 80.1 μmol/L at follow-up. Under rheumatology guidance, hydroxychloroquine 200 mg once daily was added for immunomodulation to prevent UCTD progression, with a recommendation to monitor anti-SSA/SSB antibody titers, rheumatoid factor, and complement levels every 3 months for early detection of disease progression (**Supplementary Table 2**). At 6-month follow-up, renal function remained stable, with no new UCTD-related manifestations (**Figure 4**). Family screening identified the patient’s son as a hemizygous GLA mutation carrier, and early systematic evaluation and ERT initiation were recommended.

**Figure 4:**
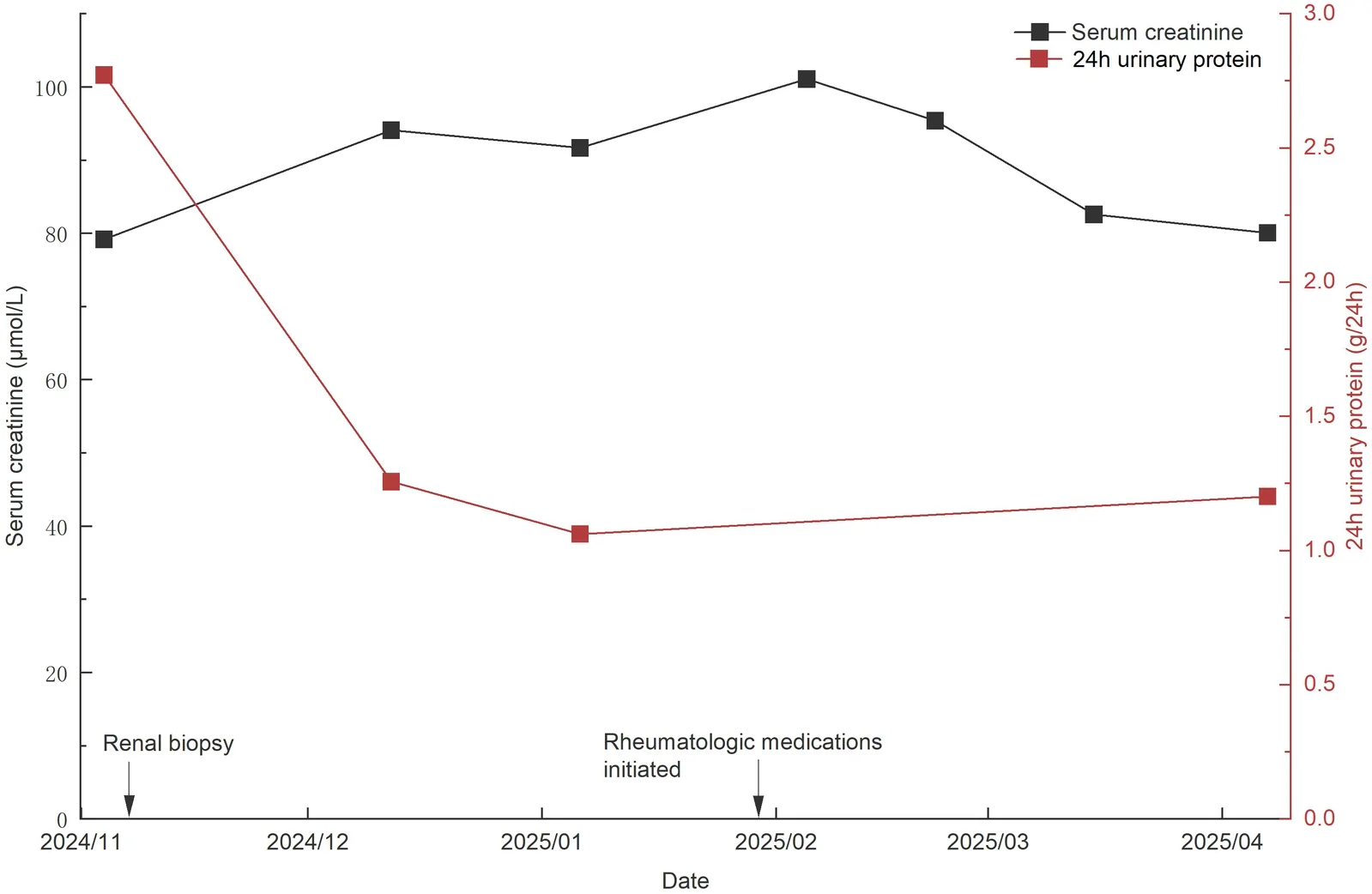
Dynamic changes of serum creatinine and 24-hour urinary protein during follow-up. The left y-axis represents serum creatinine (μmol/L), and the right y-axis represents 24-hour urinary protein excretion (g/24h). The black line with square markers indicates serum creatinine levels, and the red line indicates 24-hour urinary protein levels. Black arrows denote key clinical events: renal biopsy and initiation of rheumatologic medications.

The patient declined ERT after detailed counseling primarily due to financial constraints, as ERT is not fully covered by medical insurance in China (annual cost approximately 100,000–200,000 RMB), and expressed a preference for optimized conservative management with regular follow-up. The planned follow-up regimen includes renal function and proteinuria monitoring every 3 months, annual echocardiography and brain MRI for Fabry-related organ involvement, and rheumatology follow-up every 3–6 months with autoantibody titer monitoring. Should renal function decline or new systemic Fabry manifestations emerge, ERT will be reconsidered. The patient’s son will undergo systematic Fabry disease evaluation and early ERT initiation if indicated.

## 3 Discussion

We report a rare case of concurrent biopsy-proven FD, IgAN, and well-characterized UCTD in a heterozygous female with normal plasma α-Gal A activity. To the best of our knowledge, this is the first case describing the triple comorbidity of these three conditions, which expands the clinical spectrum of FD-associated immune disorders. While the coexistence of FD, IgAN, and UCTD in this patient raises the possibility of shared immunopathogenic mechanisms, we emphasize that a single case report cannot establish a causal relationship, and the following discussion should be interpreted as a biologically plausible hypothesis rather than a proven conclusion.

The core clinical takeaway of this case lies in the substantial diagnostic challenge of occult FD in heterozygous female patients. The patient carries the pathogenic GLA missense variant c.593T>C (p.Ile198Thr), a well-documented variant with marked phenotypic heterogeneity that may manifest as classic multisystem FD, late-onset mild disease, or even an asymptomatic carrier state (8, 9). According to the American College of Medical Genetics and Genomics/Association for Molecular Pathology (ACMG/AMP) guidelines (10), this variant is classified as Pathogenic. This classification draws on four lines of evidence: the variant has been reported in multiple unrelated patients with Fabry disease in the Human Gene Mutation Database (HGMD) and ClinVar (PS4); it resides within a critical functional domain of the α-Gal An enzyme (PM1); it co-segregates with disease in the family, as the proband’s son carries the same variant in hemizygous form (PP1); and the patient’s phenotype—renal involvement with pathognomonic zebra bodies on electron microscopy—is highly specific for Fabry disease (PP4).

Due to random X-chromosome inactivation (lyonization), the patient retained partially preserved α-Gal A activity, with both circulating enzyme levels and lyso-Gb3 falling within normal reference ranges, and lacked classic systemic FD stigmata such as neuropathic pain, hypohidrosis, and angiokeratoma (2). As a result, FD was not clinically suspected prior to renal biopsy, and the diagnosis was only triggered by incidental identification of zebra bodies on electron microscopy. In this case, zebra bodies were localized exclusively within podocytes, with no definite inclusions in glomerular endothelial cells, mesangial cells, tubular epithelial cells, or vascular smooth muscle cells; tubular epithelial cells showed vacuolar degeneration with increased lysosomes but no definitive zebra bodies. This restricted distribution is consistent with the mild phenotype of heterozygous female Fabry patients, as Gb3 deposition typically begins in podocytes and may remain limited to this cell type in early or mild disease, particularly in females with X-chromosome inactivation mosaicism (11). Drug-induced pseudomyelin bodies were considered unlikely given the absence of amiodarone exposure and the fact that hydroxychloroquine was initiated only after biopsy (11, 12). This diagnostic trajectory reinforces that routine electron microscopy during renal biopsy is irreplaceable for detecting occult FD, particularly in female patients with unremarkable family history and nonspecific clinical presentations.

Beyond diagnostic implications, the coexistence of dual glomerular pathologies in this patient raises the possibility of a non-random association between FD and IgAN, potentially mediated by Gb3-driven immune dysregulation. However, we emphasize that this remains a biologically plausible hypothesis based on a single case, and a causal relationship cannot be established. Epidemiologically, the majority of reported FD-IgAN overlap cases originate from East Asia and predominantly affect female patients, which may suggest a non-coincidental pattern but could also reflect referral and ascertainment biases. From a mechanistic perspective, several lines of evidence from the literature are consistent with a potential link between Gb3 accumulation and IgA-mediated glomerular injury. First, Gb3 has been shown to act as an endogenous damage-associated molecular pattern that can engage Toll-like receptor 4 (TLR4) and activate the NF-κB pathway in innate immune cells and renal mesangial cells (3). Second, this innate immune activation may upregulate B-cell activating factor (BAFF) and a proliferation-inducing ligand (APRIL), which could theoretically promote B-cell class switching to IgA and enhance production of galactose-deficient IgA1 (Gd-IgA1), the core pathogenic effector molecule of IgAN. Third, activated mesangial cells may upregulate IgA receptors such as transferrin receptor, potentially facilitating the capture and deposition of circulating Gd-IgA1-containing immune complexes. Molecular mimicry between the glycosyl moiety of Gb3 and nephritogenic glycosphingolipids has also been proposed as a complementary mechanism, though this hypothesis remains to be functionally validated (6). Clinically, the observation that mesangial IgA deposition may decrease after ERT in some FD patients provides indirect support for a potential role of Gb3 accumulation in IgAN development (13), but this evidence is limited by the small number of reported cases and the lack of controlled studies. In our patient, serum Gd-IgA1 levels were not measured, and therefore the proposed Gb3-TLR4-BAFF axis could not be directly validated. Whether the FD-IgAN overlap in this patient represents a causally linked phenomenon or simply the co-occurrence of two relatively common renal conditions in an at-risk individual remains an open question that requires investigation in larger cohorts.

Consistent with previous reports that up to 57% of FD patients harbor autoantibodies including ANA and antiphospholipid antibodies (4), our patient presented with high-titer ANA, anti-SSA positivity, and moderate dry eye syndrome, meeting diagnostic criteria for UCTD. Gb3 has been reported to engage TLR2 and TLR4 on dendritic cells and monocytes, potentially activating downstream MyD88/IRF signaling and driving excessive secretion of type I interferons and BAFF (14). If validated *in vivo*, excess BAFF could breach peripheral B-cell tolerance, allowing autoreactive B cells to escape clonal deletion and differentiate into autoantibody-producing plasma cells, potentially accounting for the emergence of ANA and anti-SSA. In addition, α-Gal A deficiency may impair lysosomal degradative function, disrupt autophagic flux, and reduce the clearance of apoptotic debris, leading to accumulation and enhanced presentation of self-antigens that could theoretically amplify autoimmune responses. These interconnected mechanisms, if confirmed, could create a pro-autoimmune immune microenvironment that might manifest as UCTD or progress to overt connective tissue disease over time, and could potentially explain the dissociation between severe serological abnormalities and mild glandular involvement observed in this patient. The concurrent UCTD in this patient raises a parallel question of whether Gb3-mediated immune dysregulation may extend to systemic autoimmunity. However, we acknowledge that these mechanisms have not been directly tested in our patient, and the high prevalence of autoantibody positivity in the general female population (particularly ANA, which is positive in approximately 10–15% of healthy women) means that the co-occurrence of UCTD and FD could be coincidental. Nevertheless, given the increasing recognition of immune dysregulation in FD, we suggest that routine autoantibody screening and long-term monitoring for connective tissue diseases may be considered in FD patients, particularly those with autoimmune serological abnormalities, though this recommendation requires further evidence to support its clinical utility.

From a clinical management perspective, ERT is the standard disease-specific treatment for FD, and the oral pharmacological chaperone migalastat offers an alternative for patients with amenable GLA variants. However, the p.Ile198Thr variant is not migalastat-amenable, and the patient declined ERT due to financial constraints (15, 16). Our patient’s current regimen focuses on optimized renoprotection for IgAN (combining sacubitril/valsartan, dapagliflozin, and finerenone) and hydroxychloroquine immunomodulation for UCTD. Stable renal function and absent UCTD progression at 6-month follow-up indicate that even when FD-specific therapy is deferred, active management of concurrent immune-mediated glomerular disease and systemic autoimmune abnormalities is critical for preserving organ function. In addition, family screening identified the patient’s son as a hemizygous GLA carrier, who faces a substantially higher risk of multisystem involvement; early ERT initiation is recommended to reverse subclinical organ damage.

Admittedly, several limitations of this study should be acknowledged. First, as a single case report, it cannot establish a definitive causal relationship between FD, IgAN, and UCTD, and the proposed shared immune pathways remain hypothetical. Second, the follow-up duration is relatively short, and long-term changes in renal function and autoimmune disease activity require continued observation. Further large-scale clinical cohorts and functional mechanistic studies are needed to confirm the pathogenic links between lysosomal storage disorders and immune-mediated diseases. Third, no direct *in vitro* functional assay was performed for the p.Ile198Thr variant, and protein expression levels of α-Gal A in renal tissue were not assessed by Western blot or immunohistochemistry; only plasma enzyme activity was measured, which may not reflect tissue-level protein expression. Fourth, serum Gd-IgA1 levels and BAFF/APRIL concentrations were not measured, precluding direct validation of the proposed Gb3-TLR4-BAFF pathogenic axis in this patient.

In summary, this case describes the rare triple overlap of FD, IgAN, and UCTD in a female patient with normal α-Gal A activity, whose diagnosis relied on routine electron microscopy and genetic testing. The coexistence of lysosomal storage disease, immune-mediated glomerulonephritis, and systemic autoimmunity is consistent with a biologically plausible hypothesis of Gb3-driven immune dysregulation as a potential shared pathogenic pathway, which requires further validation in larger clinical cohorts and functional studies. For patients with glomerular disease accompanied by atypical features and autoimmune serological abnormalities, occult hereditary metabolic disorders should be included in the differential diagnosis. Early renal biopsy with electron microscopy, timely genetic testing, and integrated management of concurrent comorbidities are key to improving patient outcomes.

## Data Availability

The original contributions presented in the study are included in the article/**Supplementary Material**. Further inquiries can be directed to the corresponding author.
