# Supplementary material for: Case Report: Concurrent Fabry disease, IgA nephropathy and undifferentiated connective tissue disease in a female with normal enzyme activity
Source: Front Immunol. 2026 Sep 17;17:1945437. doi: 10.3389/fimmu.2026.1945437 (PMC13627442; doi:10.3389/fimmu.2026.1945437)
Supplement: Supplementary file 1 [file Table1.docx]

# **S**upplementary Table 1: Initial laboratory investigation upon admission

| Items | Value | Normal range | Units |
| --- | --- | --- | --- |
| Blood biochemical indicators | | | |
| Serum creatinine | 79.2 | 41-73 | μmol/L |
| Urea | 5.44 | 2.6-7.5 | mmol/L |
| Uric acid | 306 | 155-357 | μmol/L |
| Serum albumin | 34.3 | 40.0-55.0 | g/L |
| AST | 30.2 | 13.0-35.0 | U/L |
| ALT | 14.7 | 7.0-40.0 | U/L |
| Blood tests | | | |
| Leukocyte | 10.17 | 3.5-9.5 | ×10^9/L |
| Hemoglobin | 125 | 115-150 | g/L |
| Platelet | 352 | 125-350 | ×10^9/L |
| Electrolyte |  |  |  |
| Potassium | 5.14 | 3.5–5.3 | mmol/L |
| Sodium | 138.1 | 137–147 | mmol/L |
| Chlorine | 107.5 | 99–110 | mmol/L |
| Phosphorus | 1.27 | 0.85–1.51 | mmol/L |
| Calcium | 2.13 | 2.11–2.52 | mmol/L |
| Magnesium | 0.83 | 0.75–1.02 | mmol/L |
| Cardiac function | | | |
| NT-proBNP | 303.0 | 0-450 | pg/ml |
| Myohemoglobin | 37.9 | 0-61.5 | ng/mL |
| CKMB | 0.61 | 0-0.38 | ng/ml |
| Troponin I | <0.012 | 0-0.034 | ng/mL |
| Electrophoresis | | | |
| Albumin | 53.49 | 55.19-69.98 | % |
| α1 globulin | 3.81 | 2.68-5.03 | % |
| α2 globulin | 11.78 | 4.93-10.19 | % |
| β globulin | 14.55 | 6.70-15.57 | % |
| Γ globulin | 16.37 | 9.81-19.81 | % |
| Alb:Globulin | 1.15 | 1.23-2.33 | % |
| Immune-related indicators | | | |
| IgA | 3.18 | 1.0-4.2 | g/L |
| IgG | 8.06 | 8.6–17.4 | g/L |
| IgM | 1.91 | 0.5–2.8 | g/L |
| C3 | 1.12 | 0.7–1.4 | g/L |
| C4 | 0.31 | 0.1–0.4 | g/L |
| CRP | 0.48 | 0-1.0 | mg/L |
| RF | 5.4 | 0-14.00 | IU/mL |
| ASO | 5.55 | 0-200 | IU/mL |
| ESR | 53 | 0-20 | mm/h |
| Serum β2-microglobulin | 3.94 | 0.70-1.80 | mg/L |
| Coagulative fuction | | | |
| PT | 11.40 | 9.0-13.0 | s |
| APTT | 26.0 | 21-33 | s |
| D-dimer | 0.36 | 0.00-0.50 | mg/L FEU |

**Supplementary Table 2: The patient's past and current medications**

| Medication | Dose | Duration | Indication |
| --- | --- | --- | --- |
| Past medications |  |  |  |
| Traditional Chinese medicine (Abelmoschus manihot, Pueraria lobata, Poria cocos) | Not documented | ~3 years (intermittent) | Self-administered for renal protection |
| Current medications |  |  |  |
| Sacubitril/valsartan | 50 mg once daily | Since Nov 2024 | Hypertension, renoprotection |
| Dapagliflozin | 10 mg once daily | Since Nov 2024 | Proteinuria reduction |
| Finerenone | 20 mg once daily | Since Nov 2024 | Proteinuria reduction |
| Atorvastatin | 10 mg once daily | Since Nov 2024 | Hyperlipidemia |
| Hydroxychloroquine | 200 mg once daily | Since Dec 2024 | UCTD immunomodulation |
| Branched-chain amino acid supplementation | 2.52 g three times daily | Since Nov 2024 | Nutritional support |
